# Organization of the St. Louis Dental Society

**Published:** 1857-04

**Authors:** 


### ARTICLE V.

### *Organization of the St. Louis Dental Society.*

At a meeting held December 11th, 1856, at the office of Drs. Dunham & Hale, at which were present Drs. Dunham, E. Hale, sr., Isaiah Forbes, J. B. McKellops, A. Blake, C. M. Forbes, E. Hale, jr., Levett, Nichols, Samuels, Hinson, Comstock, Leslie, of this city, and T. Phillips, of La.

Dr. Dunham was called to the Chair, and A. M. Leslie appointed Secretary, pro tem.

It being the unanimous sense of the meeting that a city society should be organized, a committee was appointed to prepare and present at a subsequent meeting, rules for the government of the Society.

At a subsequent meeting the Society was fully organized by adopting a simplified Constitution and By-laws, and by the appointment of permanent officers.

The chief features of these laws, are:

This Society shall be called "The St. Louis Dental Society."

"The acting members must be residents of the city or county of St. Louis." "It shall be the duty of the members of this Society, in rotation, to present at each regular meeting some case or specimen from their own practice," upon which each member present is required to present his views.

The officers elect of the Society, are, *President*, S. DUNHAM; *Vice-president*, A. BLAKE; *Treasurer*, G. H. PERRINE; *Secretary*, A. M. LESLIE; *Executive Committee*, H. BARRON, J. FORBES, G. H. PERRINE.

Since its organization this Society has taken steps, well calculated to give permanency to it. A room has been rented and furnished by the association for its convenience. Here it is hoped that it will shortly set up a dental cabinet, which by the liberality of its members, and others, will be an object of interest to their brethren, visiting the city.

We may here say that members of the profession passing through the city are freely invited to its meetings: the members taking pleasure in forming the acquaintance of their brethren from abroad.

The regular meetings are held the first Tuesday in each month. Its place of meeting is at No. 79 Market street, over the Dental Depot of A. M. Leslie.

The Society has just perfected a fee-bill which will govern the members in their charges, producing uniformity. It affords the operator liberal reward for his labor, but is not above the rates charged here previously by the leading operators.

One subject of interest was discussed by the Society at one of its meetings, viz. "What is the best method of folding foil?"

proposed by Dr. Isaiah Forbes. His method was to lay two, three, four, five, or six sheets on the top of each other, and cut them in strips as wide as the length of the cylinder he desired. These strips are rolled on a fine broach; keeping the edges parallel. The varied number of sheets give the cylinders different diameters. When preparing a quantity he puts on fine silk gloves, to avoid soiling the gold.

Dr. Spalding also uses cylinders, and folds his foil on a cushion, doubling the leaf on itself until the thickness and width desired is obtained. He divides the sheet into various sized pieces by cutting the book and foil together.

Dr. Dunham uses cylinders, and folds on a cushion, which is the general mode of folding, among the members.

Dr. J. Forbes, at a subsequent meeting, stated that he had found great advantage from placing paper between the shears and foil, when cutting it up. He considered Dr. Spalding had imparted to him a valuable hint.

The subject for discussion at next meeting is: "Is it best to anneal foil after being prepared for the cavity?"

Altogether, we may say we anticipate great practical good from this organization.

The members of this Society will receive no students for a less time than two years. The preceptor is required to pay a fee into the treasury of the Society for each student, which entitles said student to the benefits connected with the Society. Steps have been taken to form a library for the use of the students and others.

The following gentlemen have signed the Constitution:

Isaiah Forbes, C. W. Spalding, Henry Barron, Aaron Blake, S. Dunham, Jas. Wise, G. H. Perrine, Geo. H. Silvers, C. M. Forbes, I. J. Nichols, F. Hinson, A. M. Leslie, H. J. McKellops, David L. Levett, Edward Hale, jr., W. A. Jones, John H. Fairbank, H. E. Peebles.

On motion of Dr. Perrine,

*Resolved*, The Secretary be requested to prepare a synopsis of the minutes of the Society for publication.

